# A Novel Customizable Datamart and Tableau Dashboard to Monitor Multiple Enhanced Recovery After Surgery Programs: Development and Validation Study

**DOI:** 10.2196/82472

**Published:** 2026-02-11

**Authors:** Sunitha Margaret Singh, Susannah Oster, Efrat Bolze, Aaron Sasson, James Nicholson, Elliott Bennett-Guerrero

**Affiliations:** 1 Department of Perioperative Surgical Services, Stony Brook University Medical Center, 101 Nichols RoadStony Brook, NY, United States, 1 6314442166, 1 6314442907; 2 Department of Anesthesiology, Stony Brook University Medical Center, Stony Brook, NY, United States; 3 Enterprise Analytics, Stony Brook Medicine Information Technology (SBMIT), St. James, NY, United States; 4 Department of Surgery, Stony Brook University Medical Center, Stony Brook, NY, United States; 5 Department of Orthopedics, Stony Brook University Medical Center, Stony Brook, NY, United States

**Keywords:** data monitoring, enhanced recovery, enhanced recovery after surgery, ERAS, perioperative outcomes, quality improvement, web platform

## Abstract

**Background:**

Enhanced recovery after surgery (ERAS) programs bundle evidence-based interventions to standardize care, expedite recovery, and improve outcomes. As ERAS programs have expanded, it has become clear that a major challenge is monitoring the compliance of bundle elements and outcomes to feedback performance to stakeholders and guide changes. Manual data abstraction is onerous and not feasible. Reliance on receiving new reports from busy health system IT groups is challenging. Therefore, we sought to address this unmet need at our hospital by developing a novel ERAS Datamart system.

**Objective:**

Our objectives were to develop a novel Datamart and Tableau dashboard to (1) enable continuous analysis of data, harvested directly from the electronic medical record (EMR), measure compliance and outcomes, and (2) enable end users (e.g., an ERAS coordinator) to create reports customized based on surgical procedure types, requested data variables, and custom date ranges.

**Methods:**

After “buy-in” from hospital leadership and other stakeholders, data metrics were identified and categorized according to phase of care, that is, preoperative, intraoperative, and postoperative. A multidisciplinary team reviewed *International Classification of Diseases, Tenth Revision* procedure codes to capture EMR data for patients undergoing ERAS procedures. IT was given a master list with metric names, definitions, and screenshots of the discrete field in the EMR to assist with building the metrics. Validations of the novel Datamart were done against known ERAS patient populations maintained by the surgery clinic.

**Results:**

The Datamart and Tableau dashboard has been built, is functional, and contains over 17,000 patients across 5 ERAS service lines: colorectal (n=1742), joint replacement (n=4235), surgical oncology (n=941), bariatric (n=1130), and cesarean section (n=9390). Currently, 56 metrics spanning the perioperative period have been validated across these populations. Reports can be tailored according to patients, time frames, and metrics. If desired, patient-level raw data can be exported for statistical analyses. Two use cases (total joint replacement and surgical oncology ERAS programs) are presented showing how the Datamart can be used.

**Conclusions:**

Discrete fields within an EMR can be successfully captured into a novel Datamart and visualized using a custom Tableau dashboard for providing stakeholder feedback, facilitating quality improvement analyses, and auditing pathways.

## Introduction

Enhanced recovery after surgery (ERAS) programs have transformed perioperative care by implementing evidence-based interventions that aim to standardize patient care and management, decrease resource utilization, expedite recovery, and improve patient outcomes [1-3]. The success and efficacy of ERAS programs are most likely achieved through the implementation of a comprehensive approach that bundles care for patients undergoing elective surgery, encompassing approximately 20 care elements [1,4].

It is increasingly recognized that as ERAS programs increase in size, it is very challenging to monitor and track bundle elements to feedback performance and guide outcomes. Traditional methods usually rely on manual data abstraction, frequent reports generated by hospital IT systems, or the use of third-party data warehouses. As ERAS programs grow in size and complexity, manual data abstraction becomes impractical due to time demands, error risk, and challenges with real-time analysis. Basic data points, such as length of stay, are easier to track, but capturing complex metrics, such as total opioid use (e.g., oral morphine equivalents), is often not feasible. Reliance on an IT report strategy is typically limited by very long delays in obtaining data reports from hospital IT workers who are usually burdened with many requests. The use of third-party data warehouses, for example, ERAS Interactive Audit System (EIAS), offers an alternative but raises concerns about data security, control, costs, system downtimes, and limited flexibility [5]. To address the above limitations, our institution created a novel dynamic Datamart dashboard.

## Methods

### Overview

Stony Brook University Hospital is a tertiary care academic medical center on Long Island, New York. Its first ERAS program (lumbar spine fusion) began in 2016, and an additional 9 ERAS programs were subsequently added. As the program grew, this revealed the unmet need for how to efficiently capture and monitor compliance and outcomes across a large number of patients.

As described in more detail below, the process for creating this system included: (1) leadership support and data governance, (2) validated identification of relevant patients to be included in the Datamart, (3) metric identification and validation, and (4) Tableau visualization as the user interface.

### Leadership Support and Data Governance

Under an institutional quality assurance program, a guideline was developed to map the creation of a novel Datamart and Tableau dashboard and govern the data extracted. To prioritize this effort, a value statement was presented to institutional leadership. This statement provided background information on the institution’s ERAS programs, highlighted their prior success, and outlined the intended purpose of the Datamart and Tableau dashboard, such as monitoring and improving compliance, reducing errors associated with manual data abstraction, and limiting frequent report requests made to IT.

After approval, a Global ERAS Data Governance plan was conceived with policies and procedures for protecting and using ERAS data. These included how the data would be stored and protected, who would have access to data, and how data would be managed (e.g., requests for aggregate and patient-level reports, quality assurance (QA) analyses, and institutional review board–approved research projects). The Global ERAS Data Governance plan was subsequently signed by applicable departmental chairpersons to ensure data analysis was conducted in accordance with institutional standards and to protect against breaches of protected health information.

### Identification of Relevant Patients

#### Choice of *International Classification of Diseases, Tenth Revision* Codes Methods

We considered several possible strategies for identifying relevant patients for a given ERAS pathway. The hospital’s operating room schedule, that is, planned surgical procedure, provides information on the “planned” procedure; however, it does not accurately reflect the “actual” surgery performed. Current procedural terminology professional billing codes were not used since the hospital’s IT department did not have direct access to them. Therefore, we decided to use the *International Classification of Diseases, Tenth Revision* (*ICD-10*) Procedure Coding System (PCS) since this was feasible and these codes are believed to be accurate as they are used for hospital billing purposes.

To ensure the accurate identification of the ERAS patient population, the team collaborated closely with surgical leads from each ERAS pathway. Surgical procedures were sent to the coding department to identify the *ICD-10* PCS associated with specific procedures. The coding department supplied the leading 4 digits of all *ICD-10* PCS for the specified procedures. These digits encompass the section, body part, root operation, and where relevant, the body part of the given procedure. This preliminary list was forwarded to the IT department, which then appended the remaining digits of the *ICD-10* PCS, corresponding to the approach, device, and qualifier, for the procedures performed. Subsequently, a multidisciplinary team reviewed the complete *ICD-10* PCS to ensure the accurate capture of electronic medical record (EMR) data for patients undergoing ERAS procedures. This task required careful attention due to the complexities and overlaps within *ICD-10* PCS across multiple procedure types.

To ensure a comprehensive approach, a strategy was developed that included both *ICD-10* procedure codes and additional criteria, such as surgical case procedure name and other associated details (eg, associated *ICD-10* PCS, ambulatory surgical center vs main operating room, surgeon). This established a robust, multistep process for accurately pinpointing the desired patient population. This method ensured that only patients who received care associated with an ERAS pathway were included, enhancing the precision and reliability of the data captured for monitoring and analysis.

#### Population Validation Methods*  *

Next, we validated the accuracy of using these *ICD-10* procedure codes to identify the desired patient population. This validation aimed to identify instances where incorrect patients (non-ERAS patients) were erroneously included or, at the other extreme, ERAS patients were missing (i.e., not included). Admit type was then utilized to refine the patient population based on the urgency of admission, categorized as urgent, emergent, or elective. Since ERAS patients almost always fall into the “elective” category, this refinement allowed for an additional method for validating patient populations. Exception reports were generated and scheduled for automated reporting of patients who met the *ICD-10* PCS criteria but were admitted urgently or emergently. These records were then cross-referenced with the EMR to verify their eligibility for inclusion in the Datamart, ensuring only accurate ERAS populations were maintained. Several validations of the Datamart data were then performed by comparing the dataset against known ERAS patient populations maintained by the surgery clinic or stored in a REDCap (Research Electronic Data Capture) database.

### Metric Identification and Validation Methods

ERAS programs usually include many (e.g., ≥20) best practice elements. For example, the use of nonopioid analgesics, such as acetaminophen and nonsteroidal medications, is prioritized to minimize opioid use [6]. Since there are no national benchmarks for these programs to identify metrics of interest, key stakeholders (e.g.surgeons, anesthesiologists, hospital quality department personnel) were engaged. Metrics were selected, defined, and aligned with institutional interest, key performance, and patient safety indicators, and the American College of Surgeons National Surgical Quality Improvement Program (ACS NSQIP) by these stakeholders. We also sought to identify metrics that would be broadly applicable to most elective surgical procedures since the proposed Datamart system would be used for all ERAS programs.

Given the variations in documentation that can occur across an institution, it was crucial to investigate how and when an EMR field was completed. For example, nursing staff across different surgical specialties might record specifics related to urinary catheterization in separate discrete fields within the EMR. Additionally, there could be discrepancies in other charting practices as well, such as documenting ambulation as the “number of steps” taken versus “number of laps” taken. Metrics were validated with each ERAS population to identify discrepancies and ensure that the discrete field identified could be applied to the majority of surgical populations. This validation of metrics was carried out in multiple phases (e.g., 6‐10 metrics at a time with each population) to alleviate the burden of mass validation. Metric validations were performed against known ERAS populations using manual chart review to ensure accurate data extraction into the Datamart from discrete EMR fields.

Once finalized, the metrics were categorized according to phase of care: preoperative, intraoperative, postoperative, and discharge. IT was provided with metric names, definitions, and screenshots of the discrete fields in the EMR to assist with building the metrics. To compartmentalize the data in the Datamart, these phases were bucketed into 7 categories, encompassing various areas of care. These categories included patient characteristics, preoperative care, operating room, postoperative fluid, postoperative multimodal analgesia, postoperative opioid, and postoperative patient experience.

### User Interface (Tableau Visualization) Methods

Tableau was chosen as the software for enabling a web-based dashboard user interface due to its on-demand filtering options and Health Insurance Portability and Accountability Act–compliant capabilities. An open query was established between our institution’s EMR relational database and the Tableau dashboard. This setup allowed automated data extracts from the EMR to the Tableau dashboard according to the desired export rate. Multiple viewpoints and designs were trialed through various builds created by IT. Essential filtering options, such as specified time frames, surgical specialty, surgical procedures, and other specific metrics, were established and selected based on their relevance and informativeness for patients receiving care related to ERAS.

### Ethical Considerations

This project was conducted as part of an institutionally approved quality improvement/quality assurance initiative aimed at optimizing perioperative care processes and monitoring ERAS program performance. In accordance with our institution’s policies on human participants protections, quality improvement/quality assurance activities that are designed solely for internal program evaluation are not considered human participants research.

## Results

Results are presented in the same sequence of events (1-4) as described in the Methods section.

### Leadership Support and Data Governance

An ERAS data governance guidance plan was established. The document outlined the appropriate use of the ERAS data warehouse, including storage, protection from data breach and leak of protected health information, access to data, and ensuring data analysis is in accordance with institutional standards. Data access is managed by a data access group. Requests for data must be submitted in writing to the data access group for review and approval. Data can be used for quality and research scholarly activities.

### Validation of Patient Selection Using *ICD-10* Procedures Codes

Initially, 50 metrics were trialed with *ICD-10* PCS for ERAS colorectal procedures. The patient population yielded from these codes returned many patients, more than 2-fold, compared to the known ERAS colorectal population. Preliminary validations revealed the need to (1) exclude surgery types such as emergent or urgent, (2) exclude overly broad or ambiguous *ICD 10* procedure codes, and (3) screen for potential inclusion of the planned surgical case procedure. After validating the colorectal population, the initial metrics were tested in the surgical oncology and joint replacement ERAS populations. We then backfilled data to 2015 to include pre-ERAS populations.

After final validations, the Datamart (last 6.5 y) contains more than 17,000 patients, consisting of colorectal (n=1742), surgical oncology (n=941), joint replacement (n=4235), bariatric (n=1130), and cesarian section (n=9390). In addition, the Datamart contains over 3000 archived cases from 2015 to 2018.

### Metric Identification and Validation

Over 100 metrics of potential interest, spanning the perioperative period and including patient characteristics, were identified. Several of these metrics represent the same metric measured at different time points over several days, such as peak pain on postoperative day (POD) 0, peak pain on POD 1, and peak pain on POD 2. The metrics were categorized according to the phase of care: preoperative, intraoperative, and postoperative. Currently, there are 56 metrics (demographics, surgical and anesthesia care, and postoperative endpoints) in the Datamart Textbox 1.

Textbox 1. **Current metrics active in the Datamart.**

1. Patient characteristics
  - Patient age, mean or median
  - Body mass index, mean or median
  - Diabetes diagnosis, #/%
  - Current smoker, #/%
  - Chronic opioid use (regular opioid use as listed on home medications), #/%
  - Associated diagnosis code, cancer, #/%
  - Associated diagnosis code, inflammatory bowel disease, #/%
  - Actual procedure completed, #/%
2. Preoperative care
  - Preprocedural bowel prep, #/%
  - Preprocedural oral antibiotics, #/%
  - Total functional status score (sum of activities of daily living score on the day of surgery in the preoperative area), mean or median
  - Hemoglobin A_1c_ within 90 days (closest value prior to surgery start time), mean or median
  - Hemoglobin within 30 days (closest value prior to surgery start time), mean or median
3. Operating room
  - Laparoscopic procedure, #/%
  - Rectal procedure, #/%
  - Length of surgery, minutes, mean or median
  - Total intravenous (IV) fentanyl, mcg, mean or median
  - Received epidural or spinal, #/%
  - Intraoperative crystalloid IV fluids, mL (sum of normal saline, lactated ringers, and D5W), mean or median
4. Postoperative opioid
  - Day of surgery (DOS): oral morphine equivalent, mg, mean or median
  - Postoperative day (POD) 1: oral morphine equivalent, mg, mean or median
  - Postoperative day (POD) 2: oral morphine equivalent, mg, mean or median
5. Postoperative multimodal analgesia
  - Day of surgery
    - Oral acetaminophen, #/% of patients receiving any amount
    - IV acetaminophen, #/% of patients receiving any amount
    - IV or oral nonsteroidal anti-inflammatory drug (NSAID), #/% of patients receiving any amount
  - Postoperative day 1:
    - Oral acetaminophen, #/% of patients receiving any amount
    - IV acetaminophen, #/% of patients receiving any amount
    - IV or oral NSAID, #/% of patients receiving any amount
    - Multimodal agents, # of agents received, mean or median
  - Postoperative day 2:
    - Oral acetaminophen, #/% of patients receiving any amount
    - IV acetaminophen, #/% of patients receiving any amount
    - IV or oral NSAID, #/% of patients receiving any amount
    - Multimodal agents, # of types of agents received, mean/median
  - Postoperative day 3:
    - Oral acetaminophen, #/% of patients receiving any amount
    - IV acetaminophen, #/% of patients receiving any amount
  - Total IV acetaminophen (DOS through POD 3), mg, mean or median
  - Total oral acetaminophen (DOS through POD 3), mg, mean or median
6. Postoperative fluid
  - Day of surgery
    - Net input and output, mL, mean or median
    - Total input, mL, mean or median
  · Postoperative day 1:
    - Net input and output, mL, mean or median
    - Total input, mL, mean or median
7. Postoperative patient experience
  - Postoperative patient-controlled analgesia use, hours, mean or median
  - Peak pain score on day of surgery, mean or median
  - Peak pain score on postoperative day 1, mean or median
  - Peak pain score on postoperative day 2, mean or median
  - Postoperative urinary straight catheterization on day of surgery, #/%
  - Postoperative urinary straight catheterization on postoperative day 1, #/%·
  - Postoperative insertion of urinary catheter (insertion occurs more than 2 hours after surgery stop or removal in operating room), #/%
  - Postoperative duration of urinary catheter, hours, mean or median
  - Time to first flatus or bowel movement, hours, mean or median
  - Postoperative duration of nasogastric tube, hours, mean or median
  - Postoperative insertion of nasogastric tube (insertion occurs more than 2 hours after surgery stop or removal in operating room), #/%
  - Delta creatinine (peak value within 72 hours postoperatively minus preoperative value), mean or median
  - Postoperative length of stay, days, mean or median
  - Discharge opioids (oral morphine equivalents) prescribed, mg, mean or median

Unless specified otherwise, units of measurement for continuous variables are mg and categorical end points are yes/no.

### Tableau Visualization

#### Overview

The Datamart dashboard was designed to incorporate a user-friendly interface, allowing users to easily generate customizable reports. It offers tools to filter data by patient population, time frames, and specific metrics, enabling a focused analysis of ERAS outcomes (e.g., compare before and after implementation outcomes, track changes in protocols over specific time frames).

**Figure 1.**
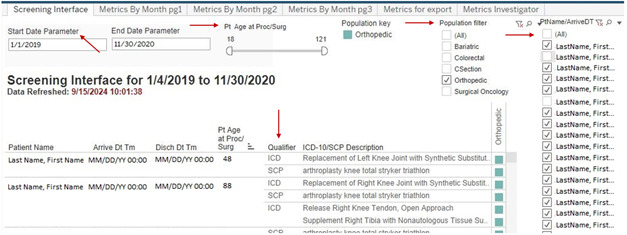
User interface (screening). User interface depicting screening options. The user can select time frames, patient age range, and surgical population. The user is presented with the patient name, arrival and discharge date and time, patient age on day of surgery, qualifier for inclusion in the Datamart (*International Classification of Diseases, Tenth Revision* [*ICD-10*] *Procedure Coding System* [PCS] and/or surgical case procedure), and qualifier description. Patients can be selected or deselected on the right. Hovering over the colored square yields additional screening data (see Figure 2).

**Figure 2.**
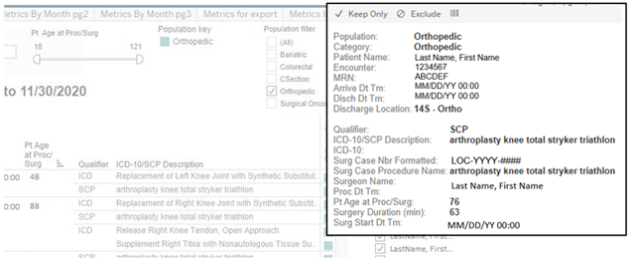
User interface (related screening). Additional screening information is available to the user. Hovering over the colored square next to the *International Classification of Diseases, Tenth Revision* (*ICD 10*)/surgical case procedure (SCP) description “pops out” information related to the selected case. This includes discharge location, surgical case number, surgeon name, and length of surgery.

The user is first presented with a screening interface where patient population, time frames, and age parameters can be defined (Figure 1). By hovering over the colored square, the user can also review certain patient and surgery-specific information, such as associated *ICD-10* PCS, surgeon, and date of surgery (Figure 2). Patients can be deselected from the population if, for example, erroneous classification of an emergency trauma patient as an elective case.

**Figure 3.**
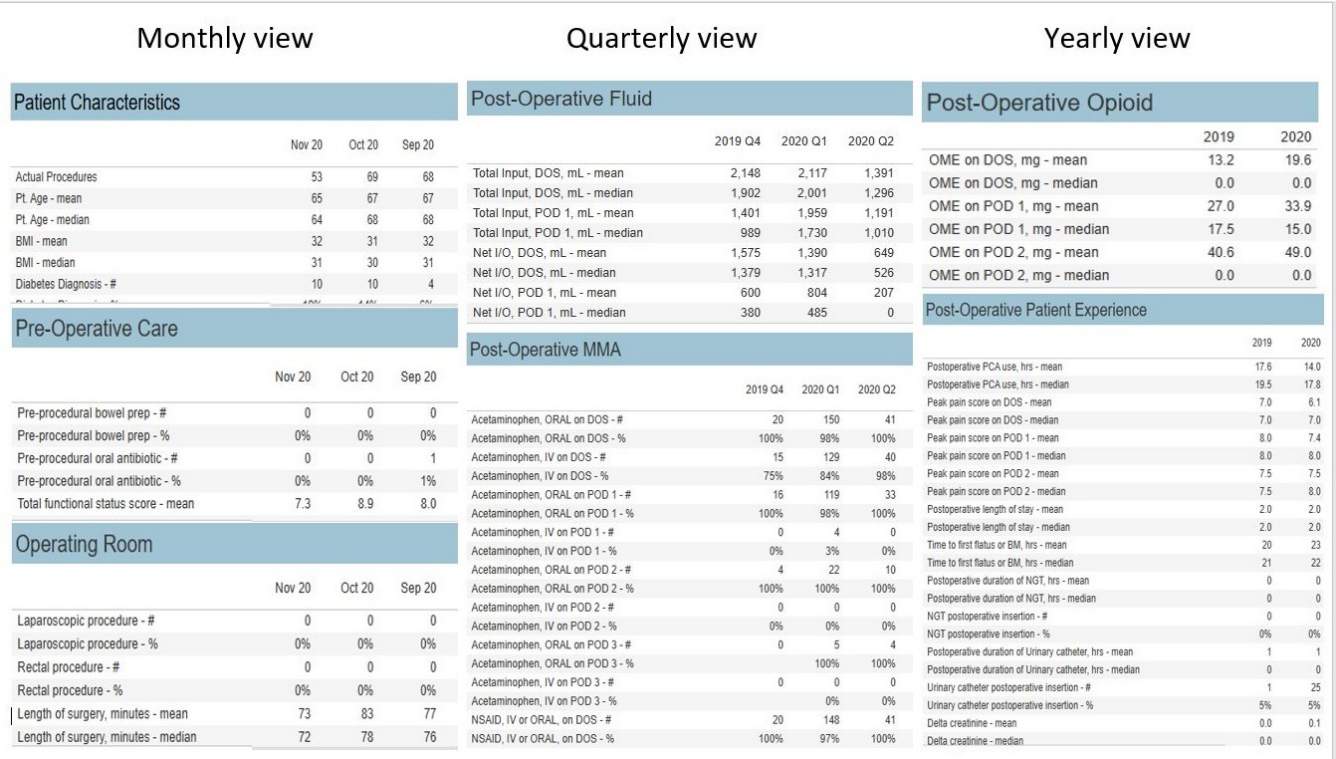
User interface (metrics). After procedures have been selected for review, the user can determine the metrics for analysis. Metrics can be viewed weekly, monthly, quarterly, or annually. Individual elements, such as opioid administration or fluid management details, can also be isolated for deeper analysis. DOS: day of surgery; IV: intravenous;nasogastric tube; MMA: multimodal analgesia; NGT: NSAID: nonsteroidal anti-inflammatory drug; OME: oral morphine equivalent; PCA: patient-controlled analgesia; POD: postoperative day.

On subsequent screens, the user can determine the metrics for analysis and how to view the data (Figure 3). Filters can be customized to focus on specific metrics or patient outcomes. For example, to focus on total acetaminophen use, one can view more details, such as “total IV acetaminophen on post-op day 1 (mean or median values).” Individual elements, such as opioid administration or fluid management details, can also be isolated for deeper analysis. Additionally, data can be displayed in weekly, monthly, quarterly, or yearly summaries and saved and exported as tables.

For further in-depth analyses, for example, inferential statistical analysis, users can export patient-level raw data in either comma-separated value or Excel format. These data can be exported in a deidentified manner, safeguarding against the unintentional disclosure of protected health information when exiting the secure Datamart dashboard. By using these features, the Datamart permits users to analyze ERAS metrics with precision, adapt to different populations, and investigate trends and outcomes.

#### Use Case Examples: ERAS Total Joint Replacement and Surgical Oncology

**Figure 4.**
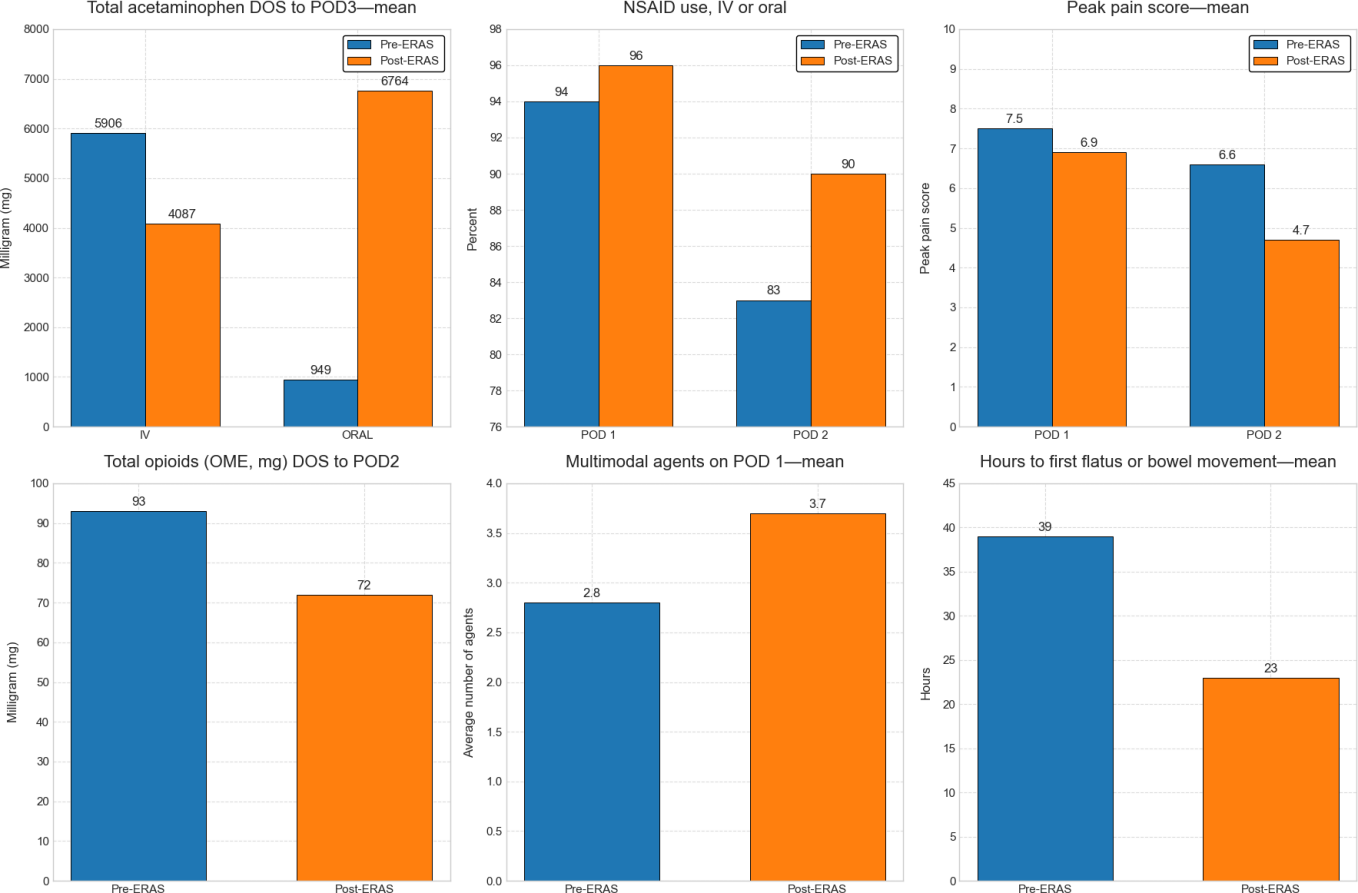
Enhanced recovery after surgery (ERAS) total joint replacement program: impact on pain. The Tableau dashboard does not display error bars for continuous variables; these require the export of patient-level raw data for statistical analysis, which is a capability of the Datamart. As ERAS pathways are primarily a quality assurance (QA) initiative, we report mean and median values, as their similarity suggests a roughly normal distribution without major outliers. DOS: day of surgery; IV: intravenous; NSAID: nonsteroidal anti-inflammatory drug; OME: oral morphine equivalent; POD: postoperative day.

Using total joint replacement as a use case example, we sought to compare patient care prior to the initiation of ERAS (pre-ERAS n=693 cases) and 12 months post-ERAS implementation (n=563 cases). Figures4  5 show the impact of an ERAS program on pain using the data harvested from the Datamart, notably an apparent improvement in several pain-related metrics. Of note, the Tableau dashboard does not calculate or show error bars for continuous variables; the calculation of these requires export of the raw data for statistical analysis. Therefore, we opted to present mean and median, since if they are similar, it suggests that the data are normally distributed and the mean value is not inflated due to a few large outliers. Therefore, for most day-to-day QA purposes, the presentation of mean and median is sufficient, but for more rigorous quantitative analysis, for example, inferential statistics for hypothesis testing, the export of patient-level raw data allows that capability.

**Figure 5.**
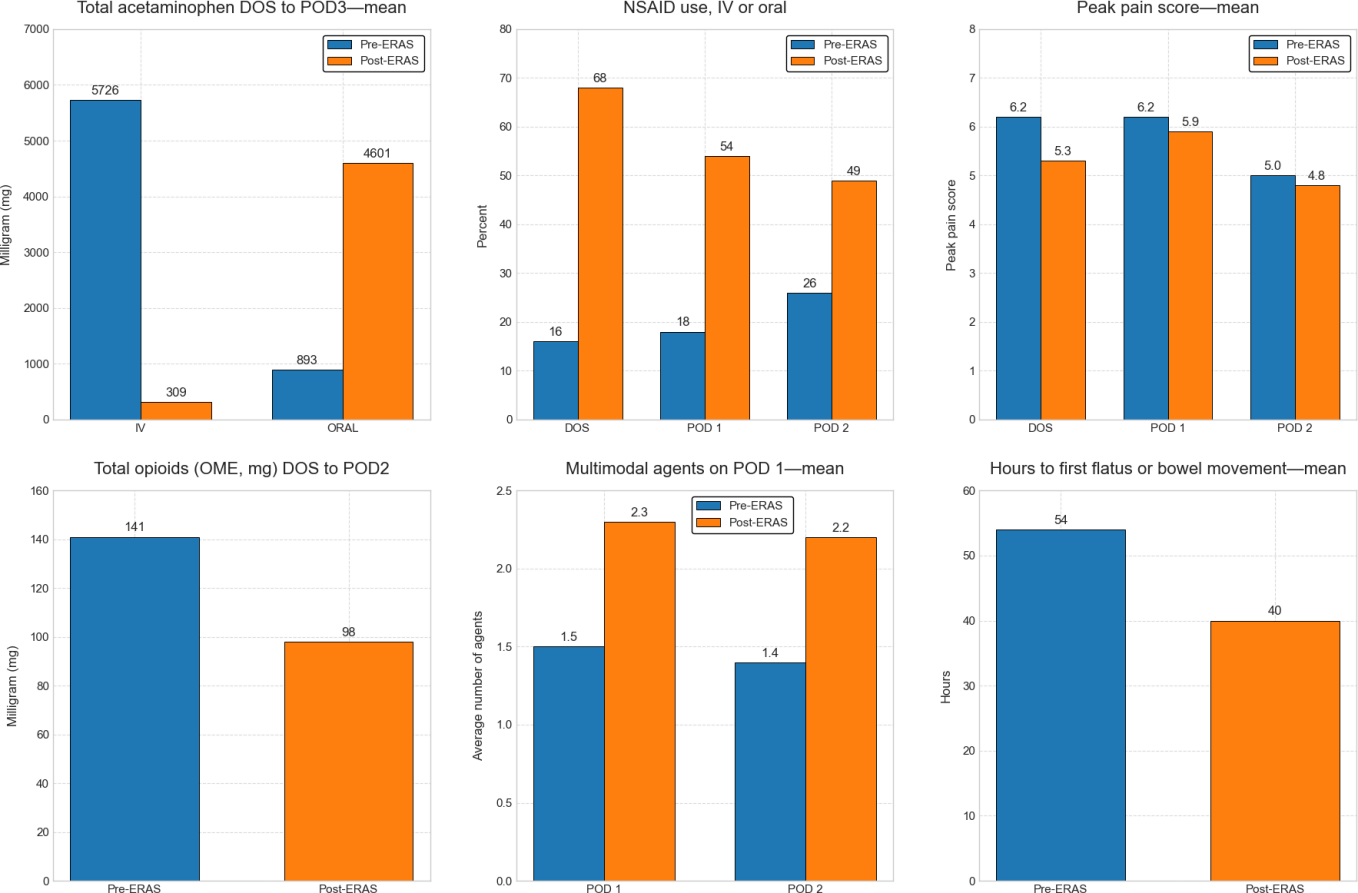
Enhanced recovery after surgery (ERAS) surgical oncology program: impact on pain. The Tableau dashboard does not display error bars for continuous variables; these require export of patient-level raw data for statistical analysis, which is a capability of the Datamart. As ERAS pathways are primarily a quality assurance (QA) initiative, we report mean and median values, as their similarity suggests a roughly normal distribution without major outliers. DOS: day of surgery; IV: intravenous; NSAID: nonsteroidal anti-inflammatory drug; OME: oral morphine equivalent; POD: postoperative day.

We noticed similar findings for our Surgical Oncology ERAS program (Figure 5).

## Discussion

### Principal Results

The novel Datamart dashboard has been a transformative tool at our hospital by enabling continuous analysis of ERAS programs and patient outcomes. By automating data extraction from the EMR, the Datamart eliminates the need for manual data abstraction, likely reducing errors and improving data accuracy. This centralized system currently serves as the primary data resource for our institutional ERAS programs, streamlining the monitoring of bundle elements and outcomes at both the individual and aggregate levels. Successful adoption required early stakeholder engagement, quarterly dashboard reviews, and review at quarterly stakeholder meetings. Barriers included initial unfamiliarity with Tableau capabilities and competing clinical priorities. These were mitigated through targeted training and dashboard use in routine quality meetings.

Key features of the Datamart include categorized metrics across perioperative phases, tailored reporting capabilities, and the ability to export data. The level of granularity of the Datamart provides actionable insights into ERAS pathway efficacy, supporting evidence-based decision-making.

### Limitations

Despite its many advantages, the Datamart has some limitations that warrant mention. As with any EMR-based form of data capture, it relies on accurate charting by clinicians, which is not infallible. For example, postoperative ambulation was not included in this iteration, as documentation practices are inconsistent. Moreover, patient-reported outcomes, such as satisfaction and functional recovery, are not currently documented in the EMR, which prevents their inclusion in the Datamart.

Accurately identifying patients on an ERAS pathway is another challenge. The Datamart relies on *ICD-10* PCS for surgical procedure identification, which can lead to the inadvertent inclusion of non-ERAS cases. For example, procedures such as hemorrhoidectomy (non-ERAS) and hemicolectomy (ERAS) share the same *ICD-10* PCS, requiring manual filtering to exclude nonrelevant cases. While procedures, such as total knee replacement or cesarean section, are easier to identify due to constrained coding options, consistent oversight is critical for accurate data classification.

An intentional 2-month lag in data import further limits real-time analysis. However, this delay ensures the accuracy of *ICD-10* coding and surgical procedure inclusion, contributing to the integrity of the dataset.

### Comparison With Prior Work

Health care auditing has significantly evolved with the advent of digital dashboards, data warehouses, and interactive audit systems. Before widespread dashboard integration, many institutions relied on manual data abstraction and semiautomated systems. Manual abstraction is cumbersome and relies on human resources to extract data from the EMR to input into a repository for analysis (e.g., REDCap, Microsoft Excel) [7-9]. Semiautomated audits (e.g. IT-generated reports), which at our institution can take 9 months or longer, add to the resource burden. Some institutions combine EMR data extraction with administrative databases (e.g., NSQIP) to build a centralized reporting structure [2,10]. However, reliance on these reports poses challenges such as delayed access to real-time data, lack of customization, and risk of system downtimes.

For some institutions with ERAS pathways, third-party data warehouses (e.g., EIAS) may be an option [5]. While they offer additional storage and analysis options, third-party warehouses present issues of data security, higher costs, and limited control over information (see Table 1). In contrast, our in-house customizable Datamart can support diverse ERAS populations and continuous improvements through iterative refinements based on user feedback and technological advancements.

**Table 1.** Comparison between the Datamart and third-party options.

Feature	Datamart+Tableau	EIAS^a^	ACS^b^ NSQIP^c^
Data control	Full institutional control over data	Vendor-controlled; limited flexibility	External benchmarking database
Customization	Highly customizable by procedure type, metrics, and time frame	Some customization possible, but limited	Standardized measures; less customizable
Real-time access	Near real-time (2-month lag for data integrity)	Typically delayed, relies on upload	Reports released quarterly or semiannually
Cost	Internal development; no licensing fees	Requires subscription or license fees	Expensive participation and data access
Data security	Remains within institutional firewall; HIPAA^d^-compliant	Data housed externally; possible security concerns	Data deidentified but externally stored
Metric flexibility	Fully institution-defined (≥56 metrics currently used)	Limited to ERAS^e^-recommended fields	Fixed set of standard metrics
Scalability	Easily scalable across services and use cases	Limited by system design and vendor	Only covers specific surgeries (e.g., colectomy)

a EIAS: ERAS Interactive Audit System.

b ACS: American College of Surgeons.

c NSQIP: National Surgical Quality Improvement Program.

d HIPAA: Health Insurance Portability and Accountability Act.

e ERAS: enhanced recovery after surgery.

While we were unable to find reports in the literature of the use of a datamart with dashboard visualization tools to support ERAS programs, these tools have been described in other types of quality, clinical, and research programs. Institutionally developed data infrastructures have been used to aggregate disparate data sources into unified repositories to support research and clinical audits [11-13]. Interactive dashboards (e.g., Tableau and Qlik) have been implemented to visualize clinical performance and facilitate quality improvement efforts [14-16]. Thus, enabling data-driven feedback to stakeholders. The Datamart combines the filtering and query capabilities of a unified data repository with the visualization tools of interactive dashboards to improve patient outcomes, enhance adherence to protocols, improve interdisciplinary communication, and support decision-making.

### Conclusions

The Datamart represents a significant advancement in ERAS program management. Unlike third-party systems, such as the EIAS or ACS NSQIP, the Datamart provides full institutional control over data, customizable metrics aligned with local priorities, and flexible reporting capabilities. Although third-party systems can be limited by fixed datasets, external data hosting, and reporting delays, the Datamart enables near real-time internal data access, tailored QA tracking, and the ability to refine metrics, as clinical needs evolve. This offers a robust, centralized, and automated approach to data monitoring and analysis without recurring licensing costs or dependence on vendor timelines. The ability to reduce manual abstraction, improve data accuracy, and evaluate intervention makes the Datamart a vital tool for enhancing perioperative care.

There are several promising directions for the enhancement and expansion of this system. Integrating predictive analytics, automated alerts, natural language processing, and machine learning into the Datamart could enable users to better anticipate complications and tailor interventions [11,12]. For example, predictive models could identify patients at high risk for ERAS noncompliance or adverse outcomes, allowing proactive adjustments to care plans. Automated alerts embedded within the dashboard could notify clinicians in real time when critical metrics fall outside expected ranges, supporting timely interventions and reducing preventable complications. Expanding the dashboard to encompass additional surgical specialties and incorporating patient-reported outcomes, through structured EMR fields or digital surveys, into the Datamart could further enhance applicability and patient-centered care. These innovations would transform the Datamart from a retrospective monitoring tool into a dynamic, decision-support platform that drives continuous improvement in perioperative care.

As ERAS programs grow in scope and complexity, the need for scalable, adaptable solutions to implementing and monitoring evidence-based care and patient outcomes is increasingly evident. This novel Datamart dashboard addresses many of these challenges while providing a foundation for ongoing innovation. Although developed within Cerner, the Datamart framework is adaptable to other EMRs, provided discrete data fields are available. Implementation requires collaboration with nursing, anesthesiology and surgical services, institutional IT, along with Tableau or similar visualization tools. Institutions seeking to improve ERAS monitoring may consider adapting the framework described here, tailoring metric section and dashboard design to their local EMR environment and clinical priorities.

## Abbreviations

ACS: American College of Surgeons
EIAS: ERAS Interactive Audit System
EMR: electronic medical record
ERAS: Enhanced Recovery after Surgery
*ICD-10*: *International Classification of Diseases, Tenth Revision*
NSQIP: National Surgical Quality Improvement Program
PCS: Procedure Coding System
POD: postoperative day
QA: quality assurance
REDCap: Research Electronic Data Capture
